# The emerging role of oxidative stress in inflammatory bowel disease

**DOI:** 10.3389/fendo.2024.1390351

**Published:** 2024-07-15

**Authors:** Peter Muro, Li Zhang, Shuxuan Li, Zihan Zhao, Tao Jin, Fei Mao, Zhenwei Mao

**Affiliations:** ^1^ Key Laboratory of Medical Science and Laboratory Medicine of Jiangsu Province, School of Medicine, Jiangsu University, Zhenjiang, China; ^2^ Nanjing Lishui People’s Hospital, Zhongda Hospital, Southeast University, Nanjing, China; ^3^ Department of Gastrointestinal and Endoscopy, The Affiliated Yixing Hospital of Jiangsu University, Yixing, China; ^4^ The Key Lab of Precision Diagnosis and Treatment in Hematologic Malignancies of Zhenjiang City, Affiliated People’s Hospital of Jiangsu University, Zhenjiang, China

**Keywords:** inflammatory bowel disease, oxidative stress, antioxidant therapy, oxidative stress markers, IBD treatment

## Abstract

Inflammatory bowel disease (IBD) is a chronic immune-mediated condition that affects the digestive system and includes Crohn’s disease (CD) and ulcerative colitis (UC). Although the exact etiology of IBD remains uncertain, dysfunctional immunoregulation of the gut is believed to be the main culprit. Amongst the immunoregulatory factors, reactive oxygen species (ROS) and reactive nitrogen species (RNS), components of the oxidative stress event, are produced at abnormally high levels in IBD. Their destructive effects may contribute to the disease’s initiation and propagation, as they damage the gut lining and activate inflammatory signaling pathways, further exacerbating the inflammation. Oxidative stress markers, such as malondialdehyde (MDA), 8-hydroxy-2’-deoxyguanosine (8-OHdG), and serum-free thiols (R-SH), can be measured in the blood and stool of patients with IBD. These markers are elevated in patients with IBD, and their levels correlate with the severity of the disease. Thus, oxidative stress markers can be used not only in IBD diagnosis but also in monitoring the response to treatment. It can also be targeted in IBD treatment through the use of antioxidants, including vitamin C, vitamin E, glutathione, and N-acetylcysteine. In this review, we summarize the role of oxidative stress in the pathophysiology of IBD, its diagnostic targets, and the potential application of antioxidant therapies to manage and treat IBD.

## 1 Introduction

Inflammatory bowel disease (IBD) is a condition that affects the digestive system and is caused by a chronic immune response. There are two main types of IBD: Crohn’s disease (CD) and Ulcerative colitis (UC). The CD is characterized by the discontinuous pattern of the ileum and colon caused by transmural inflammation, while UC occurs only in the colon and rectum. It solely affects the mucosa (1). There has been a significant rise in the prevalence of IBD in the past few years (2). According to estimates, 3.7 million people in American and European populations have IBD (3). It has been reported that IBD situations in Asia are more severe than in the West (4). Although the exact cause of IBD is still unknown, it is believed to be due to a complex interplay of genetic, environmental, and immunological factors (5). The treatment for IBD is constantly evolving, and researchers continually explore new therapies to improve patient outcomes. Currently, there is no cure for IBD, but several new medications and treatment approaches are being developed, including targeted therapies (6) and personalized medicine (7). Biologics, which target specific molecules involved in the inflammatory process, have shown great promise in the treatment of IBD (8). Additionally, stem cell therapies (9) and fecal microbiota transplantation (FMT) (10)are being studied as potential treatments for IBD. Since IBD is considered a significant global public health problem (11), There is an urgent need to explore its pathogenesis and new effective treatment options (12).

An increasing amount of evidence derived from both clinical investigations and experimental models indicates that oxidative stress signaling contributes to the development of IBD through various functional pathways. The term’ oxidative stress’, first introduced by Helmut Sies in the late 1980s (13), occurs when the production of oxidants exceeds the antioxidant defenses, leading to potential damage to biological systems (14). Oxidative stress is commonly viewed as detrimental to the body because it has the ability to harm cells, DNA, and proteins. Despite its harmful effects, some degree of oxidative stress is crucial for multiple physiological processes such as cellular signaling and immune response. Oxidative stress can damage the RNA machine involved in transcription and translation in bacteria, a critical function in bacterial survival (15). Similarly, studies indicated that protective mechanisms against oxidative stress within the bacterial cell envelope are essential for the cell’s survival (16, 17). In cancer cells, oxidative stress can act as a stimulus for inducing cell death; ROS can trigger the process of apoptosis in cancer cells by causing damage to crucial cellular components like DNA, proteins, and lipids (18). The stimulation of tumorigenesis and proliferation of cancer cells may occur due to low levels of ROS. Conversely, high levels of ROS can induce cell death (19). Therefore, oxidative stress can either be detrimental or advantageous to pathogens and cancer cells, contingent upon the concentration of ROS and the situation in which it manifests.

In IBD, evidence suggests that oxidative stress plays a crucial role in the onset and progression of the disease (20). Chronic intestinal inflammation is known to cause an overproduction of ROS and RNS, which in turn causes oxidative and nitrosative stress, respectively. These two types of stress have been linked to several human disorders, including IBD (21). Oxidative stress causes GI tract mucosal layer degradation and bacterial invasion, which triggers the immune system and leads to IBD (22). These features show that oxidative stress is a potential agent in the pathogenesis of IBD.

Over the past decades, extensive research has been conducted to understand the mechanisms underlying oxidative stress in IBD. Several studies have identified various sources of ROS and RNS in IBD, including neutrophils, macrophages, and inflamed intestinal tissue (23). The oxidative stress mechanisms may include increased ROS production, impaired antioxidant defense, biomolecule damage, mitochondrial dysfunction, epithelial cell damage, and the activation of inflammatory pathways (24–26). Considering these observations, multiple treatments that involve antioxidants, such as dietary modification, organic antioxidants, and drugs, have been suggested to decrease oxidative harm and alleviate inflammation in individuals with IBD. The prospect of oxidative stress in IBD means that antioxidant therapy may be a potential strategy for managing and treating IBD. However, further research is needed to fully understand oxidative stress’s role in IBD and determine the most effective antioxidant interventions. This review aims to summarize the mechanisms of oxidative stress, its role in the development of IBD, and the applications of oxidative stress in the diagnosis and therapeutics of IBD.

## 2 Mechanism of oxidative stress in IBD

**Figure 1:**
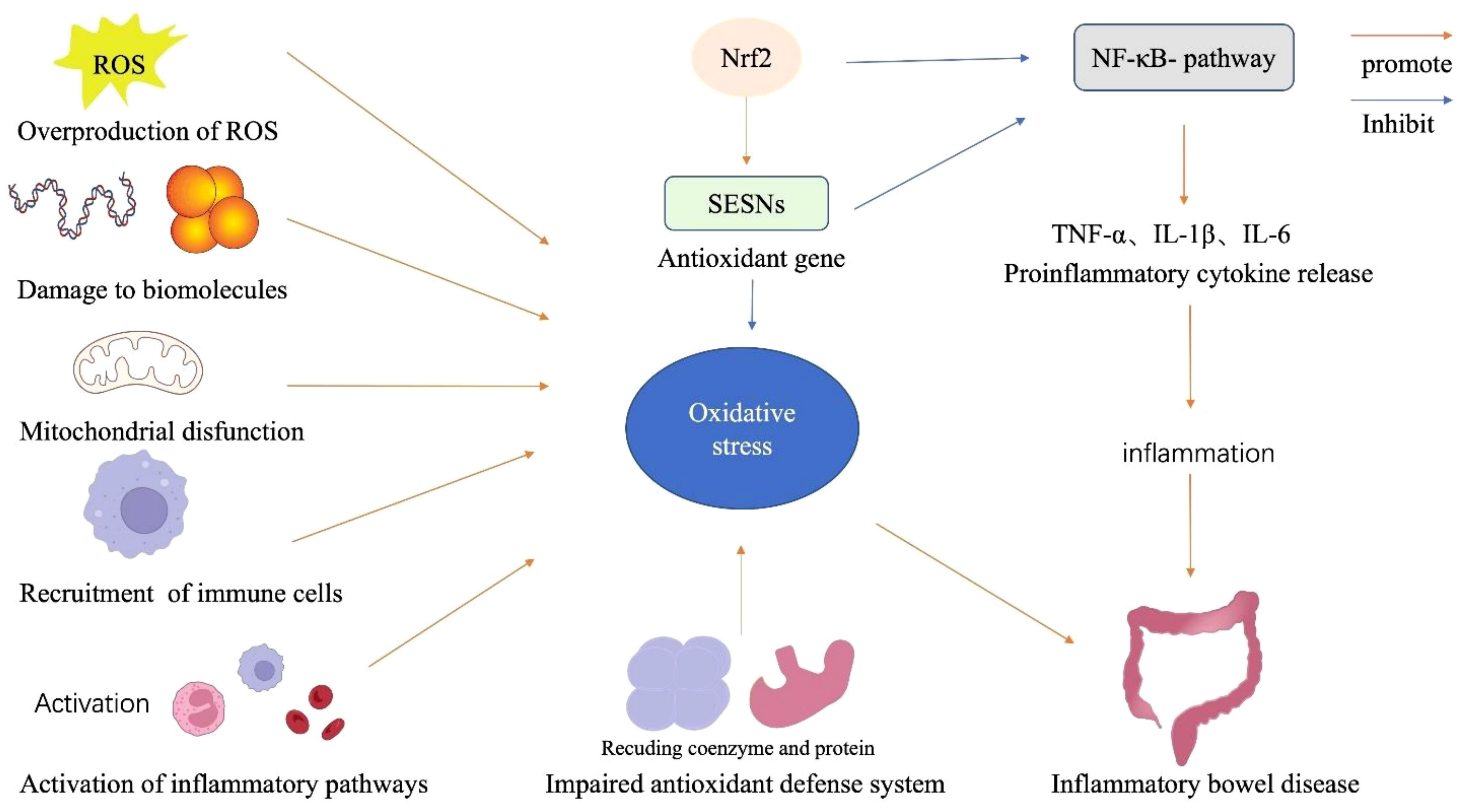
Components of the oxidative stress mechanism in IBD. The alteration of mitochondrial dysfunction, overproduction of ROS, damage to biomolecules, immune cell recruitment, and impaired antioxidant system participate in the mechanism of oxidative stress condition in IBD. Oxidative stress also triggers NF-κB activation and enhances inflammatory responses, a vital pathological component of IBD. Additionally, Nrf2 increases a variety of genes, allowing renal cells to act as antioxidants and reducing the production of cytokines and adhesion molecules that promote inflammations.

While the precise mechanisms responsible for the development of IBD remain unclear, it is widely accepted that multiple factors contribute to its etiology, including overproduction of ROS, damage to biomolecules, mitochondrial dysfunction, recruitment of immune cells, impaired antioxidant defense system, and the activation of inflammatory pathways (**Figure 1**).

### 2.1 Overproduction of ROS

One hallmark feature of IBD is the overproduction of ROS, which causes dysregulation of signal transduction, an inflammatory response, and DNA damage, all of which contribute to the progression and deterioration of the disease (27). ROS include superoxide (O_2),_ nitric oxide (NO), hydroxyl radical (-0H), hydroperoxyl radical (O_2_H), hydrogen peroxide (H_2_O_2_), and singlet oxygen (28). ROS are highly reactive molecules that occur naturally as byproducts of cellular metabolism and aerobic respiration. These compounds significantly impact physiological functions such as cell differentiation, cell signaling, cell survival, and the creation of inflammatory factors (29). Proteins, lipids, DNA, and other macromolecules are all susceptible to oxidation by ROS, which can result in chemical changes and harmful outcomes (30). Under physiological conditions, aerobic metabolism produces ROS predominantly in the mitochondria. However, excessive ROS production can disrupt cellular homeostasis, resulting in severe oxidative damage (28). In IBD, an imbalance in the redox system is caused by excessive production of ROS in colonic tissues, shown by oxidative changes to lipids, proteins, or DNA (31). The excessive production of ROS and the resulting disruption in the redox balance can give rise to oxidative stress, characterized by an increased presence of oxidative free radicals and ROS, which is closely linked to chronic inflammation and the development of metabolic diseases (24). Oxidative stress caused by ROS overproduction can contribute to the pathogenesis of IBD by damaging cellular components, activating proinflammatory signaling pathways, and impairing the intestinal epithelial barrier. For instance, high levels of ROS produced by inflammation are essential for activating macrophages in a way that further promotes inflammation (32). This activation results in the continuing release of proinflammatory mediators like IL-1β, IL-6, TNF-α, and IFN-γ, as well as increased levels of ROS (33). Hence, the interactions caused and perpetuated by the overproduction of ROS within the proinflammatory milieu lead to a self-reinforcing vicious loop that significantly contributes to the pathogenesis of IBD.

### 2.2 Damage to biomolecules

ROS can damage various cell biomolecules, including lipids, proteins, and DNA. Uncontrolled lipid peroxidation leads to harmful lipid peroxidation products. Lipid peroxidation occurs when free radicals attack and damage cell membrane lipids, particularly PUFAs (34). ROS interact with PUFAs, forming lipid radicals, which react with molecular oxygen to create lipid peroxyl radicals. The hydrophobic tails of unsaturated fatty acids receive a hydroperoxy group during lipid peroxidation. This change may affect the structural properties of biomembranes and lipoproteins by interfering with hydrophobic lipid-lipid and lipid-protein interactions or cause the production of hydroperoxyl radicals and reactive aldehyde derivates, which may result in secondary modifications of other cell components (35). In IBD, ROS, including O2−, H_2_O_2_, and •OH, can initiate lipid peroxidation by oxidizing PUFAs in the cell membranes of intestinal epithelial cells; this process leads to the formation of lipid peroxides, such as malondialdehyde (MDA) and 4-hydroxynonenal (4-HNE), which are highly reactive and cause cellular damage and inflammation (36). The elevated production of lipid peroxidation products in IBD can have several detrimental effects. For instance, 4-hydroxynonenal treatment reduces tight junction protein expression in the colon, boosts bacterial movement from the gut to circulation, and intensifies Toll-like receptor-4 signaling activation (37). Lipid peroxides damage cell membranes, disrupting the intestinal epithelial barrier and increasing permeability, allowing harmful substances and bacteria to enter underlying tissue and triggering an immune response (38). These products also activate inflammatory pathways in IBD, inducing the expression of proinflammatory cytokines, chemokines, and adhesion molecules (39).

Protein oxidation plays a critical role in the development of IBD (40). Proteins are central to cellular structure and function, and their optimal activity relies on proper folding and maintenance of sulfhydryl groups. Oxidative stress disrupts this equilibrium, leading to protein oxidation. It involves the reaction of proteins with ROS or RNS, resulting in the modification of amino acid residues, changes in protein conformation, and altered protein functions. For instance, the two most studied sulfur-containing amino acids in proteins, cysteine, and methionine, can undergo oxidation and induce changes in protein conformation (41–44). One study reported *HP1021* as a redox switch protein identified in *Helicobacter pylori*; the study shows that cysteine residues in *HP1021* are easily oxidized under cellular and laboratory conditions. This oxidation impacts the protein’s capacity to attach to DNA, and the oxidative state of the regulatory domain influences this connection with DNA (45). Hence, *HP1021* is a redox switch protein and could be a target for *H. pylori* control strategies. Besides, protein undergoes carbonylation, another form of protein oxidative modification in which ROS groups bind to specific amino acids, affecting protein stability and activity (46). It can be produced by oxidative cleavage of the Protein’s backbone or by the attack of ROS radicals on some specific amino acids in the side chains, such as lysine, arginine, proline, and threonine. Significantly, ROS can modify amino acid residues in proteins, causing structural changes and loss of enzymatic activity (47). This process can also lead to the formation of protein aggregates and disruption of normal cellular functions.

Furthermore, ROS/RNS can directly attack DNA, causing oxidative DNA damage in IBD (41). High amounts of DNA lesions can be sustained over time because cellular repair systems are compromised by prolonged exposure to oxidative stress (42). The oxidative DNA damage is more dangerous to cells because it affects the cell cycle and can lead to mutations and cancer (48). Numerous studies have shown that DNA damage plays a major role in other chronic diseases, such as various cancers, neurodegenerative diseases, inflammation/infections, aging, and cardiovascular disease (49). It has been reported that individuals with IBD exhibit elevated oxidative stress and DNA damage, particularly in lymphocytes, as observed through studies using the comet assay technique (50). The IBD patients’ DNA damage in peripheral blood lymphocytes is significantly higher, indicating that basal DNA damage may be related to insufficient antioxidant capacity and excessive ROS/RNS generation, contributing to the IBD disease’s pathogenesis.

### 2.3 Mitochondrial dysfunction

Mitochondria, with their functions in energy production, calcium homeostasis, and membrane excitability, are thought to substantially impact the pathology of IBD, as their dysfunction may initiate and advance the disease. The intestinal tract harbors abundant bacteria along with their metabolic byproducts, immune-activating molecules, molecules associated with cellular damage (DAMPs), foreign substances, and environmental pollutants capable of harming mitochondria (51). A study has reported that mitochondria in the intestinal epithelium exhibit unique protein profiles (52). Notably, these mitochondria show increased expression of ATP-binding cassette transporters, which is likely a response to the specific requirements of the gut environment (53). Additionally, it’s important to note that the gut, unlike other body parts, heavily depends on the gut microbiota for energy and the well-being of enterocytes containing mitochondria (54). Interactions with harmful bacteria such as adherent-invasive *E. coli LF82* disrupt the functioning of mitochondria in the cells lining the gut. This disrupts the balance of mitochondrial regulation mechanisms due to their strong connection with the gut’s microbial community (55). Animal models that lack genes responsible for protecting the gut’s epithelium, such as Mdr1a−/−, Irgm1−/−, and Sod2−/− transgenic mice, exhibit an increased presence of impaired mitochondria in intestinal cells and are more susceptible to experimentally induced colitis (56). Notably, 5% of the IBD genetic factors from human GWAS relate to mitochondrial balance. The leading gene associations are *SLC25A28 (mitoferrin 2), VARS (valine-tRNA ligase), and RNF5 (E3 ubiquitin ligase).* These genes control mitochondrial iron, tRNA transport, and ubiquitination (57–62). In addition, there is a connection between IBD and the *HSPA1-A, -B*, and *-L genes*, responsible for heat-shock protein 70, a key player in the mitochondrial unfolded protein response (63).

Similarly, mitophagy genes such as *PARK7* and *LRKK2* are linked to UC and CD, respectively (64, 65). Single nucleotide variations in the *C13orf31 gene*, resulting in amino acid changes in *p.C284R* and *p.I254V* in a protein of unidentified function, play a role in the development of systemic juvenile idiopathic arthritis and are associated with heightened susceptibility to leprosy and CD (66).This suggests that individuals with IBD may have an inherent vulnerability to mitochondrial dysfunction, particularly influenced by the gut environment. Additionally, intriguing connections with genes like *mitoferrin 2* hint at potentially specific pathogenic issues that remain incompletely understood. Variations in genes related to maintaining mitochondrial balance are strongly linked to the susceptibility to CD and its clinical progression. These genes include *SLC22A5*, which encodes *OCTN2, IRGM* and *UCP2* (67–69). Similarly, a study on the proteome of children with CD found that the function of mitochondria was compromised. This was particularly evident in the mitochondrial proteins responsible for detoxifying H_2_S, and this downregulation was associated with a higher disease severity (70). Again, a study documented that examinations of the mitochondria within epithelial cells of CD patients revealed disrupted and irregular mitochondrial structures, suggesting impaired function (71). These changes occur before other early inflammatory events, like modifications to tight junctions that regulate barrier function. Notwithstanding, the precise ways in which mitochondrial dysfunction affects the development of IBD are currently being studied. However, it is speculated that the impact of mitochondrial dysfunction on energy metabolism, calcium control, and membrane excitability can interfere with intestinal homeostasis, weaken immune responses, and cause the chronic inflammation seen in IBD (72).

### 2.4 Recruitment of immune cells

Within the gastrointestinal (GI) tract, the innate immune system comprises epithelial cells, neutrophils, macrophages, dendritic cells, and natural killer (NK) cells (73). In contrast, the adaptive immune system includes T lymphocytes and B cells. When activated, T lymphocytes and B cells release cytokines and antibodies (74). Under normal conditions, there is a well-regulated equilibrium in the GI mucosa between inflammatory cytokines (such as TNF-α, IL-1, IL-6, IL-8, IL-17, and IL-23) and anti-inflammatory cytokines (like IL-5, IL-10, IL-11, and TGF-β) (75). IBD impacts both innate and adaptive immunity. However, in the case of CD, it’s important to note that while adaptive immunity can perpetuate inflammation, it doesn’t trigger the initial inflammation (64). The root cause of IBD pathogenesis involves a disruption in the equilibrium between T helper (Th) cells and regulatory T cells, emphasizing the inability of regulatory T cells to function effectively.CD is characterized by inflammation driven by Th1 cells, resulting in an overproduction of IL-12, IL-17, and IL-23 (65). In contrast, UC is primarily influenced by cytokines like IL-4, IL-5, IL-10, and IL-13 produced by Th2-type T cells. In CD, the microbiota triggers the Th1 response, leading to the release of IFN-γ and TNF-α, ultimately resulting in damage to the mucosal barrier (76). In patients with IBD, the intestinal lining is frequently exposed to numerous environmental stressors, such as microbial antigens and inflammatory cytokines. In response to these triggers, immune cells such as neutrophils, monocytes, macrophages, and T cells are recruited to the inflamed mucosal tissue. This recruitment is orchestrated by chemokines, adhesion molecules, and other signaling molecules released by the inflamed tissue (77).Understanding the complex interplay between immune cell recruitment and oxidative stress in IBD is crucial for developing targeted therapies. Strategies to modulate immune cell infiltration and ROS production could potentially mitigate oxidative stress and limit tissue damage in IBD patients.

### 2.5 Impaired antioxidant defense system

Antioxidants play a major role in mitigating ROS’s effects to maintain the body’s redox balance. Antioxidants shield cells from harmful and unstable molecules by employing processes that eliminate them, thus preventing the oxidation of endogenous or non-endogenous molecules. Endogenous substances found within cells can be categorized into enzymatic antioxidants, which include superoxide dismutase (SOD), catalases (CAT), and peroxidases, or non-enzymatic antioxidants, which encompass tocopherol, glutathione, and ascorbic acid (78). Within the group of natural antioxidants, glutathione in its reduced form (GSH) primarily functions to eliminate reactive oxygen intermediates and free radicals generated during metabolic processes (79). GSH serves as a substrate for the antioxidant enzyme GPx and helps remove reactive species. It transforms into its oxidized form, GSSH, and can be converted back to GSH by glutathione reductase. Excessive free radicals can slow down this process, causing GSSH to accumulate in the cell (80). SOD is an antioxidant enzyme responsible for facilitating the conversion of the highly reactive superoxide anion (O_2_^-^) into less reactive molecules, specifically O2 and H_2_O_2_ (81). Similarly, CAT and GPx facilitate the conversion of H_2_O_2_ into water (82). Moreover, several of the antioxidant genes are recognized to have genetic variations, which can lead to differences in enzyme activity and responsiveness (83). Certain genetic variations in antioxidant enzyme genes have been linked to specific diseases, with certain genetic profiles related to a higher vulnerability to oxidative stress (84). Inadequacies in antioxidant enzymes or their compromised metabolism will increase the concentration of reactive oxygen species (ROS) and, in essence, cause oxidative stress. Research has shown that the levels of antioxidant defenses, as assessed through activities of SOD, catalase, and glutathione peroxidase, are naturally minimal within the colon and are primarily limited to the epithelial cells (85).

### 2.6 Activation of inflammatory pathways

The two main transcription factors that control cellular reactions to oxidative stress and inflammation are nuclear factor (erythroid-derived 2)-like 2 (Nrf2) and nuclear factor-κB (NF-κB) (86). The coordination of the NF-κB signaling pathway and the Nrf2 pathway plays a crucial role in driving the complex process of oxidative stress in the context of IBD.

#### 2.6.1 Nrf2 signaling

**Figure 2:**
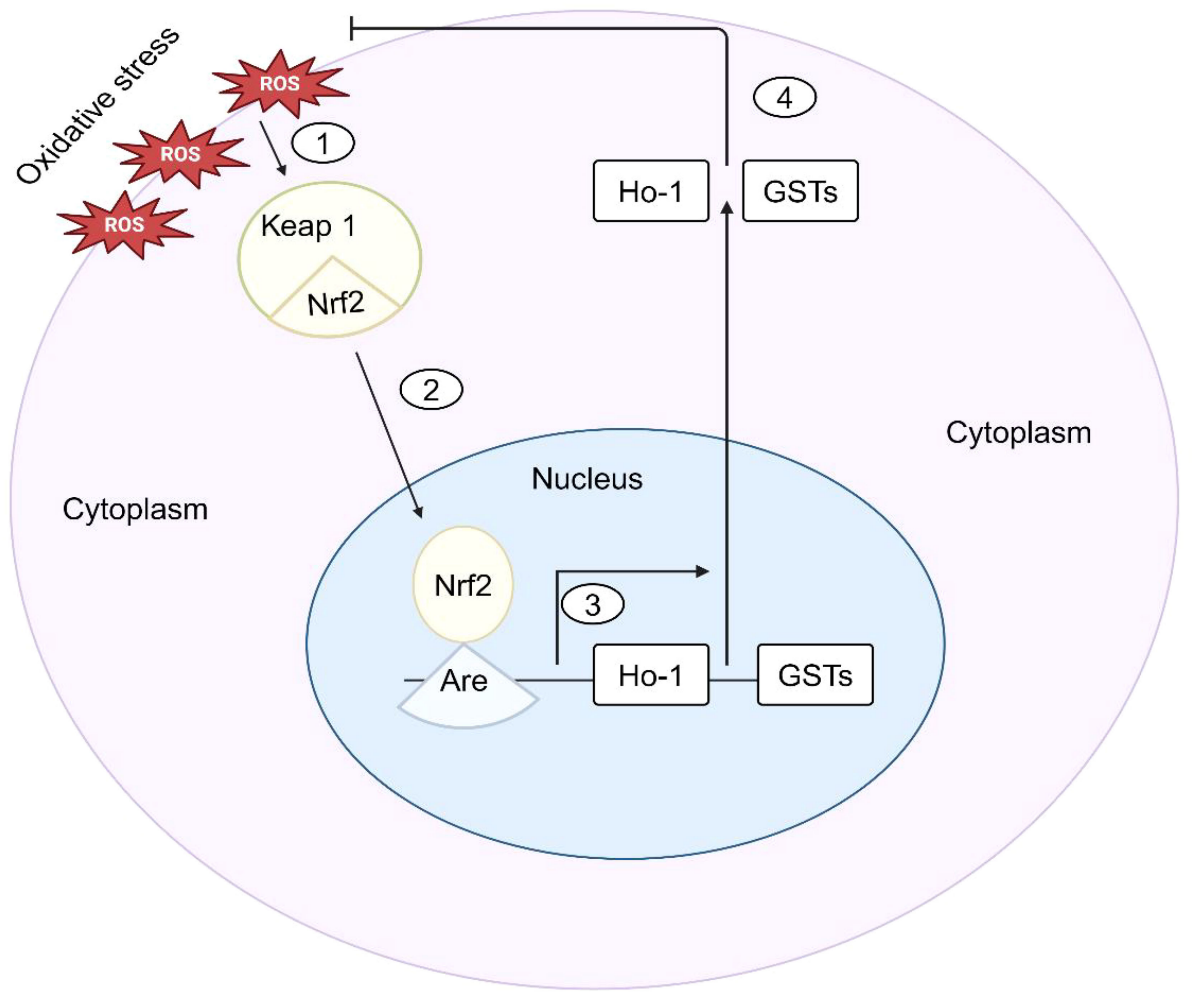
Nrf2-Mediated signaling in response to oxidative stress in IBD. (1) Upon sensing oxidative stress, cells phosphorylate Nrf2, which is normally sequestered in the cytoplasm by kelch-like ECH-associated protein 1 (Keap1). (2) The antioxidant response element (ARE) of the antioxidant genes is then bound by Nrf2 when it translocates into the nucleus. (3) HO-1 and GSTs are examples of antioxidant genes whose transcription is stimulated by Nrf2. (4) The antioxidant genes are then expressed, which prevents oxidative stress and keeps cells’ redox balance.

Nrf2 is a crucial transcription factor that plays a significant role in preserving mucosal balance by inhibiting the excessive production of ROS in IBD.Nrf2 has a dampening effect on the inflammatory response and the damage to the mucosal lining through its antioxidant actions (87). Numerous studies reveal that Nrf2 benefits cell survival and proliferation in various ways, ranging from redox homeostasis and drug/xenobiotic metabolism to DNA repair (88). Nrf2 controls the expression of several enzymatic antioxidants and results in the modulation of the levels of ROS, such as SOD, CAT, GPx, and heme oxygenase-1 (HO-1), which are vital in maintaining redox balance and cellular homeostasis. Nrf2 is a transcription factor sequestered in the cytoplasm by its inhibitor protein, Keap1, under normal conditions (89). When cells are exposed to oxidative stress, Nrf2 is released from Keap1 and translocates into the nucleus. Once in the nucleus, Nrf2 binds to antioxidant response elements (AREs) in the promoter regions of target genes. This leads to the transcription of genes involved in detoxification, antioxidation, and anti-inflammatory responses (**Figure 2**) (90). Nrf2 genes encode enzymes like heme oxygenase-1 (HO-1), NAD(P), H quinone dehydrogenase 1 (NQO1), and glutathione S-transferases (GSTs) (91). A recent study shows that upregulation of Nrf2 gene expression in a mice experiment led to increased NQO-1 protein content and activity, as well as elevated HO-1 protein content and activity in the brain, while in the liver, HO-1 activity and mRNA levels, NQO-1 activity, and protein content were augmented (92).This study reveals the tissue-specific regulation of Nrf2 signaling and downstream antioxidant enzymes in the mice, highlighting its adaptive response to varying oxygen concentrations. Similarly, in Nrf2-KO mice, there are higher levels of proinflammatory genes such as *IL-1β, IL-6, IL-8, iNOS*, and *COX-2*, and a decrease in the expression of antioxidant enzymes like hemeoxygenase-1 and GST Mu-1 (93). The activation of IER3 in the mucosa downregulates Nrf2 through the PI3K/Akt pathway, leading to decreased ROS production and apoptosis in a colitis model, which keeps Nrf2 levels low in IBD (94). Conversely, Nrf2 has been documented to reduce NOX activation and inhibit protein kinase C, consequently leading to a decrease in ROS levels and production in Nrf2-KO mice due to elevated antioxidant GSH levels, highlighting its role in mitigating oxidative stress.

#### 2.6.2 NF-κB signaling

**Figure 3:**
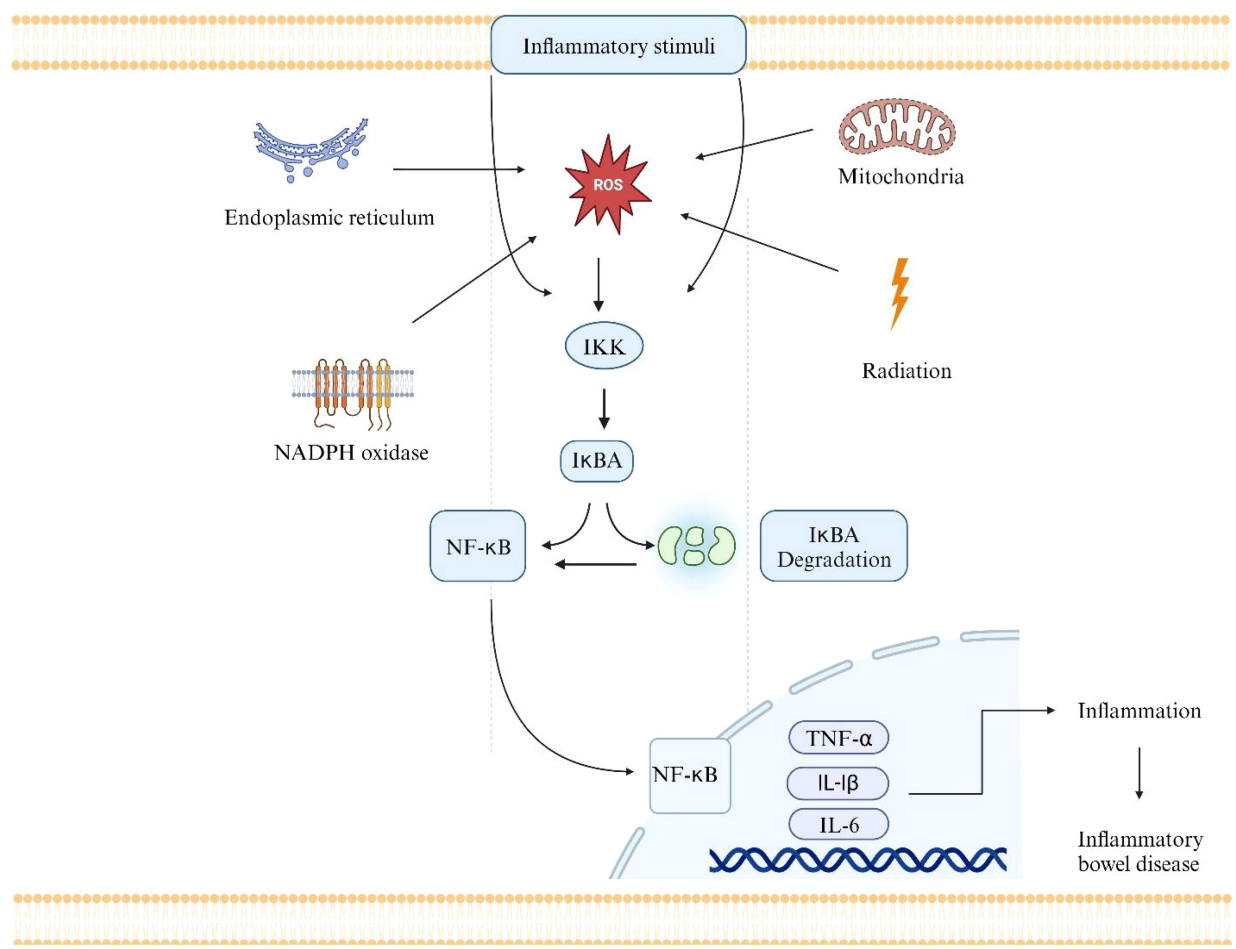
The NF-κB pathway as one of the mechanisms by which ROS can contribute to the pathogenesis of IBD. Radiation, mitochondria, NADPH oxidase, and Endoplasmic reticulum are the main sources of ROS.ROS activates the IKK complex. The IKK phosphorylates IkBA, which leads to its degradation.NF-κB is activated and translocated into the nucleus. NF-κB binds to particular DNA sequences and triggers the transcription of many different genes, including those that produce adhesion molecules, pro-inflammatory cytokines, and other inflammatory mediators (e.g., TNF-α, IL-6, and IL-1).The transcription of pro-inflammatory genes leads to the production of inflammatory cytokines and other molecules that contribute to the inflammatory response in the intestinal mucosa. Immune cells like neutrophils and macrophages are drawn to the area of gut tissue inflammation by these inflammatory signals. The influx of immune cells and the ongoing inflammation can result in tissue damage, ulceration, and the chronic inflammation that characterizes IBD.

NF-κB is a key regulator in many pathogenic processes and is abnormally active in IBD. It is a central regulator of immune and inflammatory responses (95). It controls gene transcription in immune activation, cytokine production, cell survival, and inflammation. The NF-κB pathway is a complex signaling cascade that regulates the expression of various genes involved in immunity, inflammation, and cell survival (96). It comprises a group of transcription factors linked to IκBs inhibitor proteins and remains dormant in the cytoplasm. ROS can activate the NF-κB pathway by promoting the degradation of IκBs, allowing NF-κB to translocate into the nucleus and initiate gene transcription (**Figure 3**) (97). Numerous studies have demonstrated the involvement of the NF-κB pathway in the development of IBD. NF-κB is linked to IEC homeostasis and can alter the permeability of the intestinal layer and intensify the chronic intestinal inflammation observed in the mucosa of UC and CD patients (98). Prolonged NF-κB signaling can exacerbate the persistent inflammation seen in UC and CD patients (99). In intestinal epithelial cells, the activation of toll-like receptors (TLRs) and the recognition of TNF-α by these receptors initiate the downstream NF-κB signaling pathway. This signaling pathway is also vulnerable to the effects of oxidative stress.

Additionally, the activation of NF-κB leads to increased expression of key genes, including proinflammatory cytokines like IL-6, IL-8, IL-16, and TNF-α, which contribute to inflammation. NF-κB activation upregulates genes involved in cell survival and proliferation, such as PUMA, leading to epithelial cell apoptosis and contributing to UC development (100).

## 3 Components of oxidative stress and their contributions to IBD pathogenesis

### 3.1 ROS in IBD pathogenesis

The superoxide anion (O2•−), which is produced when molecular oxygen gains one electron, is the most prevalent free radical in human tissues (101). Complexes I and III of the mitochondrial electron transport chain, which transforms 1–3 percent of total oxygen into the superoxide anion, is the primary source of O2•−in a cell (102). An additional source of O2•− is an enzymatic reaction that is catalyzed by membrane enzyme complexes known as NADPH oxidases (NOX) and xanthine oxidase (XO) (103). Out of the five isoforms of the NOX family, colon epithelium expresses NOX1 at a high level (104). Numerous studies have shown the vital role of NOX1 in IBD pathogenesis. A previous study shows the overexpression of NOX1 in large and small bowel cancers in humans (105). NOX1 expression correlates with RAS mutational status in colorectal cancer, and immunohistochemical analysis indicates overexpression in specific cancers, emphasizing its relevance as a therapeutic target in colorectal and small intestinal cancer. In IBD, analyses of biopsies from patients with CD showed increased JNK1/2 activation, as well as NOX1 and Lipocalin-2 (LCN-2) expression (106). This indicates that NOX1 might play a key role in mucosal immunity and inflammation by controlling LCN-2 expression. Again, this recent study (107) explores the impact of NOX1 loss-of-function mutations on IBD. TNF-α induces higher ROS production in NOX1-WT colonoids than NOX1-deficient ones, affecting the stem cell niche and cell. It emphasizes ROS modulation for future IBD therapies. Notably, NOX1 is vital in IBD. A recent study highlights NOX1’s involvement in peroxynitrite production induced by the microbiota. Certain NOX1 variants, such as *NOX1 p. Asn122His*, are linked to impaired gut barrier function. The research further examines the structural dimension, indicative of a crucial asparagine residue in the NOX1-p22phox complex, vital for the electron transfer process in human NADPH oxidases (108). Similarly, NOX1 facilitates the transmembrane electron transfer to two molecular oxygens, forming when activated. It has been proposed that NOX1-induced O2•− at the colon’s luminal surface Influence various processes such as bacterial virulence, expression of bacteriostatic proteins, epithelial renewal, restitution, and microbiota composition to control the intestinal innate immune defense and homeostasis (109). NOX1 and NOX4 have been linked to the pathologies associated with the hepatitis C virus as long-lasting, endogenous ROS generators (110). Besides, NOX4 has been specifically linked to oncogenic H-Ras- (H-RasV12-) induced DNA damage and senescence, suggesting a potential role in HCV-related oncogenesis (111).

Moreover, oxidative stress causes an increase in O2•− concentrations, which triggers the Haber-Weiss reaction and excessive production of the harmful hydroxyl radical (OH •). A Fenton reaction catalyzed by Fe^2+^ and H_2_O_2_ also produces the hydroxyl radical (112). Other transient metals, such as copper, chromium, or cobalt, may contribute to the generation of OH• in place of ferrous metals. These reactions become a significant source of OH• in the presence of oxidative stress conditions or when the concentration of free, unbounded transient ions rises, as in the case of hemodialysis. The OH• depolymerizes GI mucin, inactivates pyruvate dehydrogenase, an essential mitochondrial enzyme, and damages mitochondrial RNA and DNA in the GI tract (113). The perhydroxyl radical (HOO•) is another protonated form of O2•− that starts the peroxidation of fatty acids (114). Lipid peroxidation alters lipoproteins into pro-inflammatory forms, disrupts biomembranes’ fluidity, permeability, and integrity, and produces potentially hazardous byproducts (115). Furthermore, it has been demonstrated that lipid peroxidation products have carcinogenic and mutagenic qualities (116). In addition to mitochondria, peroxisomes and plasma membrane NADPH oxidases are other sources of free radicals in cells. These organelles use oxygen to produce H_2_O_2_. Catalase (CAT) converts peroxisome-derived H_2_O_2_ to water and oxygen under physiological conditions (117). On the other hand, a damaged peroxisome contributes to oxidative stress by directly releasing H_2_O_2_ (118). In Fenton and Haber-Weiss reactions, H_2_O_2_ may be transformed, along with O2•−, into the highly toxic and oxidizing OH• hydrogen peroxide (119). XO is the primary source of O2•− in the GI tract. As a result, the reaction mediated by GPx and/or CAT transforms it to H_2_O_2_.MPO uses the H_2_O_2_ neutrophil produced to create hypochlorite ions (OCl −) (120). The O2•− is a highly unstable, highly reactive, and short-lived form of ROS and reacts quickly to become membrane-impermeable. As a result, it acts close to its source, oxidizing nearby biomolecules, while H_2_O_2_ can freely diffuse across cell membranes and oxidize molecules farther away, such as pathogen membrane lipids. Aquaporin-8 (AQP8) facilitates H_2_O_2_ diffusion in GI (121). This specific aquaporin isoform is crucial in controlling H_2_O_2_ membrane permeability and signaling, making it an essential player in redox signaling processes (122). These studies (123–125)documented the involvement of AQP8 in modulating H_2_O_2_ transport through the plasma membrane, influencing redox signaling pathways associated with leukemia cell proliferation (126, 127). It’s interesting to note that enterocytes exhibit varying baseline levels of ROS. The small intestine, for instance, maintains a lower concentration of ROS, whereas the colon has a higher concentration (128). The variations in ROS generation could affect the amounts of oxidized proteins, lipids, and DNA damage, increasing the colon’s vulnerability to GI disorders at these two intestinal sites (129). Circulating XO binds to vascular endothelial cells in pathological states, causing site-specific oxidative damage to intestinal tissue (130). In IBD, a retrospective study documented XO activity concerning adverse effects from thiopurine therapy. The results indicated lower XO activity in patients experiencing adverse effects; the findings imply that monitoring XO activity might be useful in predicting and managing thiopurine-induced toxicities (131). Furthermore, O2•− is produced during a sequence of events known as “the respiratory burst” that activated neutrophils go through (132). Research has demonstrated that NOX enzymes, particularly NOX2, are involved in this process. This is because mice lacking NOX2 exhibit lower levels of oxidative burst and are less vulnerable to experimentally induced UC (133).

### 3.2 RNS in IBD pathogenesis

RNS comprise the second category of free radicals, produced as a byproduct of nitric oxide synthases(NOS) and expressed in specific intestinal submucosa and mucosal regions. Through a five-electron oxidative reaction, NOS converts arginine to citrulline and produces the nitric oxide radical (NO•) (134). Three main isoforms of NOS are inducible NOS (iNOS), which is present in various cells and tissues; endothelial NOS (eNOS), which is initially identified in vascular endothelial cells; and neuronal NOS (nNOS), which is discovered primarily in neural tissue (135). The iNOS continuously produces NO•, in contrast to the pulsative nature of eNOS. The overproduction of RNS in activated macrophages, leukocytes, and epithelial cells in the intestinal mucosa is caused by iNOS, which is only found in inflammatory tissue (136). Numerous studies have shown the involvement of NOS isoforms in IBD. According to this study (137), it has been shown that in UC, the activation of the iNOS/COX-2/5-LOX loop and increased levels of their end products, such as NO, prostaglandin E2 (PGE2), and leukotriene B 4 (LTB 4), which lead to the overproduction of free radicals and the impairment of the antioxidative system. For instance, this study found that patients with active IBD exhibited elevated mRNA expression of iNOS in intestinal biopsies, indicating increased inflammation. This suggests a specific role of iNOS in the inflammatory response associated with IBD, emphasizing its potential significance in understanding the disease’s pathophysiology (138). Similarly, in an earlier study, the up-regulation of iNOS in the intestinal epithelial cells (IECs) has been closely associated with the initiation and maintenance of intestinal inflammation in IBD, which can be potentially used as a non-invasive blood-based biomarker of IBD, as documented (139). The role of iNOS in IBD is further complicated by its relationship with cytokines and pro-inflammatory cytokines, which upregulate iNOS expression (140). Furthermore, nitrotyrosine is produced when tyrosine and NO derived from iNOS react. Research has shown that patients with UC but not collagenous colitis exhibit strong nitrotyrosine-stained epithelium linked to neutrophil infiltration (141). Additionally, iNOS is among the central downstream genes of NF-κB, but, in turn, iNOS can promote and inhibit NF-κB activity (142).

On the contrary, the Enos isoform, which is localized to the microvasculature at the submucosa–mucosa interface, catalyzes the capillary recruitment of absorptive hyperemia. The vasodilatory actions of NO• play a prominent role in this process (143). Again, the nitric oxide radical reduces leukocyte adhesion to endothelial cells and shields epithelial cells from toxicity induced by H_2_O_2_ (144). Notably, the increased expression of the eNOS gene reduces the expression of adhesion molecules in endothelial cells, mitigates colitis induced by DSS in mice, and is associated with severe cases of UC, as documented (145). This suggests that eNOS could serve as both a potential prognostic marker and a target for therapeutic intervention. Besides, the nNOS isoform also plays a significant role in the pathophysiology of irritable bowel syndrome (IBS) and other gastrointestinal disorders, including IBD. For instance, this study (146) utilized a neonatal maternal separation stress model in mice to simulate irritable bowel syndrome (IBS) and identified neuronal nitric oxide synthase (nNOS) as a novel and reliable biomarker for interstitial cells of Cajal stimulation in IBS. This is further supported by studies (147), which identified deficits in nNOS neurons in various enteric neuropathies, including those associated with IBD.

Moreover, One mechanism through which RNS contributes to IBD pathology is the formation of Peroxynitrite(ONOO-), a highly reactive oxidant formed by the reaction of NO• with O2•− (148). ONOO-is produced by cells that contain NOS enzymes, such as smooth muscle or endothelial cells, as well as by stimulated leukocytes during an inflammatory response. ONOO- can induce damage to cellular structures, including lipids, proteins, and DNA, leading to oxidative stress and further exacerbating inflammation and tissue injury (149). Research elucidates a novel HMGB1-mediated inflammatory pathway in Non-Alcoholic Fatty Liver Disease (NAFLD), revealing a redox signaling mechanism where ONOO-, formed through NADPH oxidase activation, plays a pivotal role in TLR-4 activation and cytokine release (150). The findings highlight the significance of ONOO-as a key mediator in intestinal inflammation in NAFLD. In IBD, the increased production of NO•, coupled with excessO2•−, results in elevated ONOO-levels. Again, a recent study indicates that in an animal experiment, NOX1 plays a crucial role in the production of ONOO- in the intestines (151). Similarly, the impact of ONOO-on Na-amino acid co-transporters (*NaAAcT*) in rabbit intestinal villus cell brush border membrane during chronic intestinal inflammation. ONOO- inhibits Na-alanine co-transport (*ASCT1*) by reducing its affinity for alanine and Na-glutamine co-transport (*B0AT1*) by decreasing co-transporter numbers, revealing potential mediation of *NaAAcT* alterations in inflammation (152).

### 3.3 Lipid peroxidation and lipid radicals in IBD pathogenesis

Both RNS and ROS can exacerbate lipid peroxidation. Because they are high in PUFAs, membrane lipids and lipoproteins are particularly vulnerable to oxidative damage. A hydroperoxy group is added to the hydrophobic tails of unsaturated fatty acids during lipid peroxidation. Through disruption of hydrophobic lipid-lipid and lipid-protein interactions, this change may result in structural changes to biomembranes and lipoproteins. Alternatively, it may cause the production of reactive aldehyde derivatives and hydroperoxyl radicals, which may cause secondary modifications to other cell components. Lipid peroxidation’s byproducts, such as 4-hydroxynonenal or malondialdehyde, can react with lysine amino groups, histidine imidazole groups, or cysteine sulphydryl groups to damage proteins (153). These reactions can result in the formation of adducts, which can serve as biomarkers of oxidative stress and lipid modification. LOX enzymes, which catalyze the dioxygenation of polyenoic fatty acids to form hydroperoxides, are another source of lipid radicals. 5-LOXs play a significant role in the intestines by catalyzing the oxidation of arachidonic acid. GPx subsequently reduces the hydroperoxides produced by LOX enzymes (154). It has been documented that patients with CD have higher plasma levels of lipid peroxidation products, a decreased peroxidation potential, and an oxidative low-density lipoprotein level, particularly during an active phase of the disease (155). IBD patients experience lipid peroxidation, but the cause varies based on the type of IBD. The amount of lipid peroxidation products is associated with epithelial CAT expression and neutrophilic MPO activity in UC, suggesting an H_2_O_2_-and/or OCl-mediated mechanism. In CD, lipid peroxidation is associated with mitochondrial superoxide dismutase (SOD) activity, suggesting the involvement of OH• and O2•− (156). This is further supported by the finding that SOD activity is increased during active disease and returns to normal in remission (157).Additionally, the presence of oxidative damage and the inhibition of catalase, an antioxidant enzyme, in CD patients’ immune cells further underscores oxidative stress’s role in the disease (158). These results indicate a possible involvement of lipid peroxidation and SOD activity in the IBD.

### 3.4 Cytokines and signal pathways

Reduced cytokine synthesis, which inhibits T cell and macrophage activity, may be linked to the pathophysiology of CD. According to an earlier study, intestinal tissue from CD patients exhibits a reduced expression of IL-4 mRNA, a cytokine that postpones the formation of O_2_•− in PMNS (159). Particularly in those who are genetically predisposed, external and environmental variables play a significant role in the start and progression of IBD, a complicated multifactorial illness. Similarly, IL-36, a cytokine that can induce fibrosis, is found at higher levels in fibrotic intestinal tissues of CD patients (160). Additionally, patients with CD have lower levels of antioxidative substances such as plasma ascorbic acid, α- and β-carotene, lycopene, and β-cryptoxanthin, as well as tissue GSH, which takes part in GPx-catalyzed H_2_O_2_ reduction (161). Antioxidative enzymes like GPx and SOD, however, tend to be dependent on the state of CD as well as the serum level; GPx activity is stable or reduced during CD remission and increases during active CD (157). In individuals with CD, oxidative stress occurs both locally and systemically. It is linked to the disease’s well-documented dysbiosis and unbalanced immunological response. According to a previous study, the mice models of UC and CD demonstrate that the colon’s up-regulation of GPx2 gene expression and down-regulation of aquaporin 8, which facilitates H_2_O_2_ diffusion, may protect against severe oxidative stress in IBD (162). In addition to IL-4, several other cytokines, including TNF-α, IL-1β, IL-6, and IL-8, contribute to CD (163). ROS and RNS are capable of inducing the release of these cytokines. It has been documented that patients with active CD had up-regulated NOS mRNA expression in their colonic mucosa and peripheral blood mononuclear cells (164). Similarly, it also suggested a positive correlation between NOS-derived NO• and plasma levels of IL-6, IL-17A, and IL-23 in Sjögren’s syndrome(SS), as documented (165).

**Figure 4:**
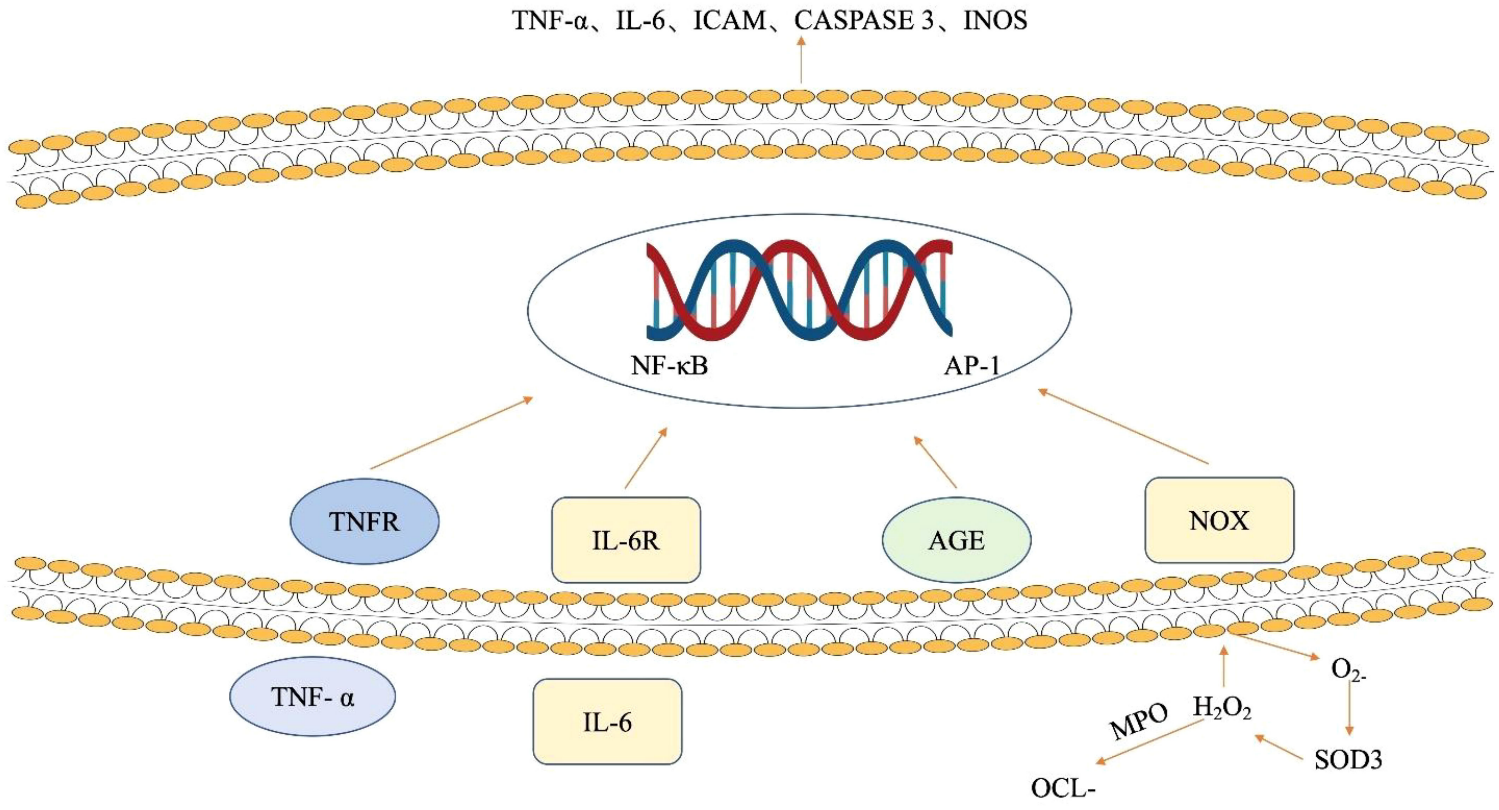
The influence of ROS and cytokines on signaling pathways in intestinal epithelial cells. NOX enzymes generate superoxide anion, elevating advanced glycation end product (AGE) in epithelial cell membranes. SOD3 converts superoxide to hydrogen peroxide, enhancing AGE content. Concurrently, NF-κB is activated by AGE, NOX, and pro-inflammatory cytokines (IL-6, TNF-α), triggering increased expression of caspase 3, ICAM, TNF-α, and IL-6 genes. Meanwhile, MAPK activation boosts AP-1 signaling and iNOS production. AGE advanced glycation end products, AP-1 activator protein 1, ICAM intracellular adhesion molecule, IL-6 interleukin 6, IL-6R interleukin 6 receptor, iNOS inducible nitric oxide synthase, NF-κB nuclear factor-kappa B, NOX NADPH oxidase, MAPK mitogen-activated protein kinases, OCl − hypochlorite ion, SOD3 extracellular superoxide dismutase, TNF-α tumor necrosis factor-alpha, TNFR tumor necrosis factor receptor.

Also, some of the environmental risk factors linked to CD may be caused by oxidative stress. The precise etiopathology of CD is still unknown, but oxidative stress is widely acknowledged to play a critical role in the disease’s pathogenesis. The aforementioned cytokines act through the mitogen-activated protein kinase (MAPK) and NF-κB signaling pathways. Aberrant NF-κB activation is implicated in the pathophysiology of IBD (166). The involvement of NF-κB and MAPK signaling pathways in IBD is shown in (**Figure 4**). NOX enzymes produce superoxide anion and other free radicals. The advanced glycation end products (AGE) content in the plasma membrane of epithelial cells is directly increased by the conversion of the superoxide anion to hydrogen peroxide by SOD3, as seen in (**Figure 4**). In summary, the NF-κB signaling pathway is activated by both AGE and NOX, as well as pro-inflammatory cytokines such as IL-6 or TNF-α. This leads to an increase in the expression of *caspase 3*, *ICAM, TNF-α*, or *IL-6 genes*. On the other hand, activation of MAPK improves the expression of AP-1 signaling molecules and increases the production of iNOS, the uninhibited source of NO. When combined, the inhibition of NF-κB or p38 MAPK may impact ROS/RNS production and reduce the generation of cytokines in patients with IBD, particularly when the disease is actively progressing (167).

Similarly, combining TLR4/NF-κB and Nrf2-ARE pathway modulation offers a comprehensive approach to managing IBD by simultaneously reducing inflammation and enhancing antioxidant defenses. For instance, a study showed Morningside from *Cornus officinalis* inhibits LPS-induced inflammation and oxidative stress in RAW 264.7 macrophages by blocking TLR4/NF-κB and activating Nrf2/HO-1 pathways (168). It reduces pro-inflammatory factors and ROS generation, and promotes HO-1 expression, suggesting its potential as an anti-inflammatory and antioxidant agent. Besides, Phosphoinositide 3-kinase (PI3K)/Akt signaling regulates cell survival and oxidative stress responses. Enhancing PI3K/Akt signaling can protect against oxidative damage in IBD. In UC mice, glutamine (Gln) reduced oxidative stress-induced injury by inhibiting the PI3K/Akt signaling pathway (169). The study showed that Gln administration improved superoxide dismutase and glutathione peroxidase activity, decreased malondialdehyde content, and ameliorated colitis symptoms and histological damage. These findings suggest that targeting oxidative stress via these molecular pathways like MAPK, TLR4/NF-κB, Nrf2, and PI3K/Akt may offer new therapeutic strategies for managing IBD.

## 4 Targeting oxidative stress in IBD diagnosis

IBD poses a significant challenge in gastroenterology due to its complex and multifactorial nature. The dysregulation of the immune system in the gut is a key factor in the development of IBD (170). Recent findings indicate that oxidative stress is crucial to the disease’s onset and progression (108). A study (171) emphasized the critical role of oxidative stress in the development and progression of IBD, as well as the production of ROS and antioxidant defense systems. Similarly, the involvement of oxidative stress in sepsis, which disrupts redox signaling, leading to molecular damage, has been highlighted (172). The dysregulation between ROS and antioxidants contributes to sepsis progression, impacting cellular function and mortality. This emphasizes the role of ROS in sepsis pathophysiology and the potential of antioxidant adjunct therapies (172). These studies collectively contribute new insights into understanding the role of oxidative stress in IBD and potential therapeutic avenues targeting ROS-mediated pathways in the management of this condition. Presently, extensive research is being conducted to investigate the significance of oxidative stress in diagnosing IBD. These studies examine the importance of oxidative stress markers as essential indicators and explore their potential utility for diagnostic applications. ROS and RNS are key components of the oxidative stress event implicated in IBD (173). These molecules, produced at abnormally high levels in individuals with IBD, exert destructive effects on the intestinal lining. This damage not only contributes to the disease’s initiation but also activates inflammatory signaling pathways, further amplifying the inflammatory cascade. Within this intricate web of events, oxidative stress markers (OSMs) such as malondialdehyde (MDA), 8-hydroxy-2’-deoxyguanosine (8-OHdG), and serum-free thiols (R-SH)emerge as valuable diagnostic targets for IBD diagnosis. MDA, an aldehyde with reactive properties formed through the peroxidation of polyunsaturated fatty acids, serves as a prominent OSM in IBD, reflecting lipid peroxidation and cellular damage and functions as a marker for cellular damage caused by free radicals (174). According to this study finding (175), there is a positive correlation between elevated lipid hydroperoxide (LOOH) levels and heightened MDA levels, suggesting MDA as a potential biomarker for lipid peroxidation and indicative of the influence of endogenous oxidative stress in individuals with CD. Numerous studies have consistently emphasized the increased levels of MDA in patients with IBD. This highlights MDA’s role as a reliable marker for assessing oxidative stress in the IBD. For instance, Elevated levels of MDA in Tunisian patients, as observed in biopsies from individuals with CD, suggest the involvement of oxidative stress in the pathophysiology of IBD (176). Similarly, these studies (155, 177–179) show that MDA levels were higher in the serum and saliva of IBD patients. This specifies that MDA can be used as a valuable marker for assessing oxidative stress in these patients, with its levels positively correlating with disease activity and inflammation. MDA plays a crucial role in diagnosing and tracking the effectiveness of treatment and the progression of the disease (175). Assessing MDA levels offers valuable information about the degree of lipid peroxidation and oxidative harm, helping guide therapeutic approaches. The substantial evidence from these studies solidifies MDA as a pertinent biomarker for oxidative stress in IBD, contributing to our understanding of disease pathogenesis and management.

On the other hand, 8-OHdG emerges as a crucial biomarker reflecting oxidative DNA damage (180). It is a modified form of guanine, one of the four nucleotide bases that make up DNA. The formation of 8-OHdG, a modified nucleoside, is a consequence of oxidative DNA damage, particularly guanine residues (181). It has been documented in a meta-analysis study and a systematic review that elevated levels of 8-OHdG in biological samples, such as urine, serum, or tissue, are indicative of increased oxidative stress (182, 183). Notably, Elevated levels of 8-OHdG in individuals with IBD have been consistently documented in research, indicating its promising role as a dependable OSM in the context of this disease (123–125, 158, 184). Similarly, a recent study revealed elevated levels of 8-OHdG in individuals with CD patients compared to those in the healthy control group (185). This suggests that 8-OhdG is a valuable indicator for evaluating oxidative stress in individuals with CD. The inflammatory milieu in IBD leads to enhanced ROS generation, causing DNA damage and subsequent 8-OHdG formation (186). Numerous studies have documented the association between elevated 8-OHdG levels and the severity of IBD symptoms, linking this marker to the progression of the disease. For instance, Assessing 8-OHdG levels provides valuable insights into the extent of oxidative damage, aiding in disease prognosis and therapeutic strategies (187). Again, elevated levels of 8-OHdG have been linked to disease severity in various conditions, including cardiovascular disease (188), chronic periodontitis (189), Huntington’s disease (190), chronic kidney disease (191), and colorectal tumors (192–194). These findings suggest that 8-OHdG may serve as an OSM for disease activity and progression in various inflammatory and degenerative diseases. Utilizing 8-OHdG as a diagnostic instrument aligns with the increasing focus on personalized healthcare and targeted therapeutic strategies in the context of IBD (195). Moreover, interventions targeting oxidative stress, informed by 8-OHdG levels, could hold promise in mitigating IBD progression. 8-OHdG stands out as a robust marker for oxidative stress in IBD, offering a molecular insight into disease pathology and potential avenues for therapeutic interventions.

Besides, R-SH consistently indicates systemic oxidative stress because they are easily oxidized by ROS, making them a reliable biomarker for oxidative stress in IBD and other diseases (196, 197). For example, a study (182) examined oxidative stress in IBD and found that R-SH levels were significantly lower in IBD patients compared to healthy individuals. These free thiols, which indicate systemic oxidative stress, showed a strong correlation with endoscopic disease activity and were more effective in distinguishing disease severity than fecal calprotectin levels. Likewise, another research (183) documented significantly lower levels of R-SH in CD and UC patients compared to controls, with these lower levels correlating with increased inflammation severity and reduced in corticosteroid-treated patients, identifying systemic thiol stress as a key marker of oxidative stress and inflammation in IBD. Additionally, a recent study (198) suggests that R-SH, an OSM, could serve as a biomarker for IBD, proving more sensitive than C-reactive protein (CRP) in detecting moderate endoscopic activity, though less sensitive than fecal calprotectin, with age and albumin levels as potential confounding factors, and indicates that R-SH may improve IBD monitoring. Again, the intervention of Leucine-rich alpha-2 glycoprotein (LRG), a serum biomarker for inflammation in IBD, has shown greater accuracy than CRP in assessing clinical and endoscopic disease activity in UC, suggesting it may be a more reliable marker for inflammation in IBD (199–201).

As such, measuring MDA, 8-OHdG,and R-SH levels provides a quantitative and qualitative assessment of oxidative stress, providing clinicians with a valuable tool in the diagnostic armamentarium for IBD. The diagnostic utility of OSMs goes beyond simple identification, extending to the monitoring of disease progression and assessing the effectiveness of treatment interventions. The correlation between OSM levels and disease severity implies that tracking these markers over time can provide insights into the dynamic nature of IBD. Overall, these three OSMs play a crucial role in the pathogenesis and progression of IBD and have been suggested to serve as potential diagnostic, differential, progression, and prognostic markers in the disease. Further research is needed to fully understand the role of OSMs in IBD and their potential as biomarkers in clinical practice.

Furthermore, Antioxidant strategies may prove beneficial in alleviating oxidative stress and mitigating the progression of IBD. Antioxidants, such as vitamin C (Vit-C), vitamin E (Vit-E), glutathione, and N-acetylcysteine, represent a potential therapeutic strategy. Vitamin C, known for its potent antioxidant properties, is crucial in alleviating oxidative stress and potentially mitigating the progression of IBD. Oxidative stress, marked by an imbalance between free radicals and antioxidants, is implicated in IBD pathogenesis. Vit-C acts as an antioxidant by scavenging free radicals, thereby reducing oxidative damage to cells and tissues (202). This helps reduce inflammation and oxidative damage, improving IBD patients’ outcomes (203). Several studies have explored the correlation between Vit-C and OSMs in various inflammatory conditions, highlighting its protective role. Combining Vit-C into the treatment regimen may offer therapeutic benefits by countering oxidative stress in IBD (204). Similarly, Vit-E, a fat-soluble antioxidant, primarily protects cell membranes from oxidative damage by interrupting the chain reaction of lipid peroxidation (205). Vit-E’s antioxidant properties combat ROS and reduce MDA, with various studies indicating a correlation between OSMs, including MDA, and the severity of IBD (206). For instance, a current study demonstrates that the combination of pentoxifylline (PTX) and Vit-E exhibits notable anti-fibrotic effects in human primary intestinal myofibroblasts (HIMFs) and murine models of IBD. This combination treatment suppresses the expression of fibrogenic markers induced by *TGF-β1*, showing efficacy in preventing colonic fibrosis. The findings suggest that PTX and Vit-E co-administration could be a promising therapeutic approach for IBD (207). The positive correlation between Vit-E levels and a reduction in oxidative stress suggests its potential as a therapeutic agent for managing IBD.

Incorporating Vitamin E-rich foods or supplements may be beneficial in supporting the treatment of IBD. Also, Glutathione, a tripeptide composed of cysteine, glutamate, and glycine, is a crucial endogenous antioxidant that plays a key role in detoxification and free radical scavenging. In the gut, glutathione is essential for maintaining the redox balance and protecting against oxidative stress-induced damage (208). The correlation between reduced glutathione (GSH) levels and OSMs, such as MDA, is explored in studies. For instance, a significant decrease in GSH levels in hypertensive patients was found, which was associated with an increase in MDA (209). Also, it has been documented that glutathione is regulated by the transcription factor Nrf2 and is vital in protecting cells from various stressors. Its forms, reduced GSH and oxidized GSSG, along with associated enzymes like GPx and GST, contribute to detoxification and redox balance, influencing cell survival under stress and impacting cancer chemoprevention and treatment sensitivity (210). Similarly, another study reveals a significant increase in postprandial reduced GSH levels compared to postabsorptive levels, emphasizing the importance of postabsorptive specimen collection for accurately assessing the basal level of reduced glutathione (211). These studies collectively highlight the importance of reduced GSH in mitigating oxidative stress and its potential as a biomarker for oxidative damage. Notably, maintaining an optimal balance of antioxidants, including glutathione, may be key in managing inflammation and disease progression in patients with IBD. Moreover, N-acetylcysteine (NAC), a precursor to glutathione, has been extensively studied for its antioxidant properties. It acts by replenishing intracellular glutathione levels and directly scavenging free radicals. Research suggests that NAC supplementation may help protect the gut from oxidative stress-related injuries and inflammation (212). Collectively, these antioxidants contribute to the overall defense against oxidative stress in the gut, preventing cellular damage and inflammation. Hence, integrating antioxidant therapies into the diagnostic framework not only adds a layer of precision to IBD management but also emphasizes the interrelation of diagnosis and treatment in the context of oxidative stress. Hence, targeting oxidative stress in IBD diagnosis emerges as a promising avenue with far-reaching implications. By understanding the intricate interplay between OSMs and disease pathophysiology, can help enhance diagnostic precision, monitor disease progression, and tailor therapeutic interventions for individuals with IBD. The integration of antioxidant therapies further solidifies the role of oxidative stress as a key player in IBD, bridging the gap between diagnosis and treatment in the pursuit of more effective and personalized patient care.

## 5 Application of oxidative stress in IBD therapeutics

Oxidative/nitrosative stress is a significant pathophysiologic aspect involved in the development and course of IBD. Since inflammatory cells secrete a large number of cytokines and chemokines, oxidative stress is triggered during inflammation, and overproduction of ROS is stimulated. In light of this, treatment strategies involving compounds with anti-inflammatory and antioxidant qualities might be considered. As IBD involves oxidative stress and inflammation, these diverse antioxidants collectively act as a shield against cellular damage, offering a multifaceted approach to treatment. The complex interplay between the endocrine system, redox balance, and oxidative stress requires understanding how hormones like melatonin, estrogen, and insulin act as antioxidants, while others like thyroid hormones and corticosteroids increase oxidative stress. Plant compounds like alkaloids and flavonoids show potential in combating oxidative stress in diabetes. Extracts from pomegranate peel and grapeseed, rich in polyphenols, are studied for their effects on ovarian cancer and female reproductive health. Nutritional antioxidants such as selenium and vitamin C are known to counter adrenal hormone-induced oxidative stress. Research supports the role of melatonin in improving testicular health and fertility by reducing oxidative stress. These findings emphasize the importance of antioxidants in managing various endocrine-related conditions (213). In exploring the potential therapeutic agents for IBD, a study reports a variety of polyphenolic substances, phenolic compounds, alkaloids, storage polysaccharides, phytochemicals, and antioxidant hormones, such as resveratrol, curcumin, quercetin, berberine, tamarind xyloglucan, sulforaphane, ginger, and melatonin (196). Synthetic antioxidants provide targeted support, while natural oxidants, derived from plant sources, contribute to a holistic and sustainable therapeutic strategy since they are used within regulations’ parameters (197). Additionally, micronutrient antioxidants, such as vitamins C and E, further bolster the body’s defense mechanisms. Moreover, adjunctive therapies such as prebiotics, probiotics, and postbiotics are also used to manage oxidative stress and help treat IBD. Prebiotics are dietary components crucial for mammalian nutrition. They can positively impact enteric diseases and oxidative stress by altering gut microbiota composition and producing short-chain fatty acids (SCFAs) (214). This can enhance immune function, improve the gut barrier, and stimulate beneficial microorganisms, potentially preventing disease and oxidative stress. Probiotics are live microorganisms that, when consumed correctly, boost health, create competition in the gut against harmful bacteria, and promote a healthier environment (215). Combined as synbiotics, prebiotics, and probiotics show promise in treating IBS by modulating microbiota, gut barrier function, immune responses, and the gut-brain axis (216). Clinical studies demonstrate their efficacy in alleviating IBS symptoms. Postbiotics mainly refer to biologically active components secreted by bacteria (217). Their advantages over probiotics include a reduced risk of infection or potential side effects triggered by the administration of viable microorganisms to immunocompromised individuals. The most important postbiotics are organic acids, SCFA, tryptophan (Trp), and bacteriocins. Understanding the synergy among these antioxidant modalities holds promise for enhancing IBD management and improving patient outcomes.

### 5.1 Natural antioxidants used in IBD therapy (polyphenols)

Examples of the phytochemical family known as polyphenols present in many plant diets are flavonoids, phenolic acids, lignans, and stilbenes. An increasing number of studies have shown that in the early stages of IBD, natural polyphenols can effectively reduce the severity of intestinal inflammation and oxidative stress (218). Diets high in polyphenols may improve the pathophysiology of conditions in which the overproduction of ROS contributes to the development of the illness (219). **Table 1** displays polyphenols and various plant-derived compounds possessing antioxidant properties.

**Table 1:** IBD treatments with natural antioxidants.

Antioxidant	Model of study	Mechanism of action	Clinical Manifestations	Ref.
Resveratrol (RSV)	BALB/C mice model.	Upregulation of *Arg1* and *Slc6a8* and downregulation of iNOS through arginine metabolism.	Reduces colitis, modulates cytokines, promotes anti-inflammation.	(220)
	BALB/c mice model.	Alleviates colitis via cytokine modulation and ANRIL-miR-34a pathway.	Reduced colitis by modulating cytokines, miR-34a, MUC2, GLNAT7, and ANRIL.	(221)
	BALB/c mice model.	Inhibition of SUMO1 and Wnt/β-catenin pathway.	Reduces colitis, modulates cytokines, promotes anti-inflammation.	(222)
	TNBS-induced colitis murine model.	Inhibition of pro-inflammatory cytokines.	Reduces inflammation, MDA levels, and increased GPX activity.	(223)
	Randomized double-blind, placebo-control.	Decrease the severity of the disease and increase quality of life.	Decrease the severity of the disease and increase quality of life.	(223–225)
Curcumin (CUR)	DSS colitis Mice model.	Stabilization of the gut-liver axis.	Improvement of DAI, colonic mucosal injury, and inflammatory infiltration.	(226)
	Primary rat VSMCs model.	Inhibition of the TLR4-MAPK/NF-κB pathways.	Reducing the overexpression of inflammatory mediators, NO production, and the activation of TLR4, MAPK/NF-κB pathways.	(227)
	DSS-induced colitis mouse model.	Inhibits NLRP3 inflammasome activation.	Mitigates colitis symptoms and reduces inflammation.	(228)
	Randomized Controlled Trial.	Decrease the severity of the disease and increase quality of life.	Higher clinical and endoscopic remission rates. Adverse events were rare.	(228, 229)
	A Randomized, Double-Blind, Multicenter Study.	Decrease the severity of the disease and increase quality of life.	No adverse events and reduction in clinical disease activity.	(230)
Quercetin (QCT)	(TNBS) induced rat model.	Weakens the clinical, morphological, and biochemical alterations via its antioxidant mechanism.	Mitigates TNBS-induced changes with antioxidant action.	(231)
	(TNBS) induced colitis model.	Eupatilin and QCT quercetin both mitigate IBD.	Reduces MPO activity, elevates GSH levels, and attenuates lipid peroxidation.	(232)
	DSS-induced colitis model.	Modulating gut microbiota and its metabolites SCFAs.	Increasing goblet cell density and mucus protein, Reducing the overexpression of inflammatory mediators, and MPO levels.	(233)
Catechines	Randomized double-blind, placebo-control.	Polyphenon E resulted in a therapeutic benefit for patients refractory to 5-aminosalicylic and azathioprine.	Active treatment remission rate. Minor side effects.	(234)
	(TNBS)-induced colitis model.	Ameliorating colitis through the NF-κB pathway.	Effective anti-inflammatory and antioxidant impact, and stabilizing mast cells.	(235)
	C57BL/6J mice model.	Inhibition of pro-inflammatory cytokine.	Improved DAI score, reduced intestinal score.	(236)
Anthocyanins	Clinical trial. (UC patients)	Decrease the severity of the disease and increase quality of life	Improved clinical symptoms.	(237)
Silymarin	A randomized, double-blinded, placebo-controlled clinical trial.	Decrease the severity of the disease and increase quality of life.	Improvement in hemoglobin level, Improved DAI score, and High remission rate.	(238)

### 5.2 Synthetic antioxidants used in IBD therapy

Synthetic antioxidants used in IBD therapy include medications, hormones, enzymes, and other biochemical substances that are presented in **Table 2**.

**Table 2:** IBD treatments with synthetic antioxidants.

Antioxidant	Model of study	Mechanism of action	Clinical manifestation	Ref.
Melatonin	Randomized clinical trial.	Decreased level of anxiety and depression.	Help sustain remission in UC patients. Steady CRP levels.	(239)
	DSS-induced mice model.	Increased antioxidant capacity. Improve oxidative stress resistance of mice with colitis.	Regulate microbial flora. Improve intestinal health.	(240)
N-acetylcysteine (NAC)	TNBS-induced colitis model.	Suppressed COX2 and E (2) (PGE (2) levels.	Reduced iNOS activity.	(241)
Modified Superoxide Dismutase (SOD)	TNBS-induced mouse model.	Recombinant Lact. Fermentum reduces oxidative stress via the NF-κB pathway.	Higher survival rate and lower DAI score.	(241)
	Pilot study.	Improved UC therapy.	Less severe side effects.	(242)
	Double-blind control trial.	Safe treatment.	Improve serum level markers.	(243)
Propionyl-L-carnitine (PLC)	Mildto moderate UC/CD patient’s study.	Improve clinical symptoms.	Decrease DAI. No side effects.	(244)
	Clinical trial	Higher safety profile. Improve clinical symptoms.	Mild digestive system adverse reaction.	(245)

### 5.3 Micronutrient antioxidants used in IBD therapy

Micronutrient antioxidants in IBD therapy include vitamins E and C, reduced glutathione, and selenium, as shown in **Table 3**.

**Table 3:** IBD treatments with micronutrient antioxidants.

Antioxidant	Model of study	Mechanism of action	Clinical manifestation	Ref.
Vitamin E	Clinical trial.	Novel therapy for mild to moderate active UC.	No side effects.	(246)
	Pilot study.	Lower infection frequency and disease severity.	Improved neutrophil count and function.	(247)
	C57BL/6 mice model.	PTX and Vit-E suppressed TGF-β1 induced expression of fibrogenic markers.	Exhibit significant anti-fibrotic effects on both human primary intestinal myofibroblasts (HIMFs) and *in vivo* IBD models.	(207)
Vitamin C	C57BL/6 and BALB/C.	Boost Antioxidant enzymes (SOD, CAT, GPx).	Lowers the expression of pro-inflammatory cytokines (iNOS, TNF-α). Lowers MDA levels.	(246, 247)
	Clinical trial.	Reduce corticosteroid dosage for disease control.	Significant improvement in clinical symptoms.	(248)
Selenium	DSS-induced mice model.	Has minimal impact on inflammatory processes and disease progression.	Alleviate inflammation and Lower disease severity.	(249)

### 5.4 Adjunctive therapies used in IBD treatment (prebiotics, probiotics, and postbiotics)

Adjunctive therapies such as prebiotics, probiotics, and postbiotics are used in managing oxidative stress and the treatment of IBS and IBD, as presented in **Table 4**.

**Table 4:** IBD treatments with adjuvant therapies.

Adjuvant therapy	Model of study	Treatment	Clinical manifestation	Ref.
Prebiotics	A Prospective Observational Study.	Oral microencapsulated sodium butyrate (BLM).	BLM supplementation appears to be a valid add-on therapy for remission in UC patients.	(250)
	A randomized, double-blind, placebo-controlled study.	scFOS	Improved rectal discomfort, IBS symptoms, and quality of life. scFOS reduced anxiety and increased Bifidobacteria in feces.	(251)
Probiotics	A pilot, randomized, double-blind, placebo-controlled study.	Lactobacillus and Bifidobacterium species.	Significantly induced remission in UC patients. Reduced stool frequency and improved biochemical markers like C-reactive protein, hemoglobin, and IL-10 levels.	(252)
	Randomized Controlled Trial.	Specific probiotics.	Significantly reduced oxidative stress (d-ROMs) and boosted antioxidant response (BAP), improving patient health safely and effectively.	(253)
	A randomized clinical trial.	Bacillus coagulans Unique IS2.	Improved GI symptoms like pain and bowel movements. Demonstrated safety and efficacy for adult IBS. No impact on inflammatory cytokines.	(254)
	DSS-induced colitis in mice.	Lactobacillus (Pediococcuspentosaceus, Lactobacillus plantarum, and Weissellacibaria).	Reduced DAI, pathological score, regulated cytokine secretion at the level of gene expression, and increased colon length. Potential treatment for IBD.	(255)
Postbiotics	Clinical trial with a randomized controlled design.	Sodium butyrate.	A significant increase in the colonic IL-10/IL-12 ratio was found within butyrate-treated patients. Rectal butyrate enemas had minor effects on inflammation and oxidative stress in UC patients.	(256)
	DSS IBD mouse model.	D-methionine (D-Met) and/or butyric acid (BA).	Reduced disease severity and suppressed inflammation-related gene expressions. Potential therapeutic for IBD.	(257)
	DSS-induced colitis model.	Heat-killed *Bifidobacterium bifidum* B1628 (HB1628).	Reduced DAI, histology scores, and pro-inflammatory cytokines, with increased IL-13. Reduced inflammation, improved gut microbiota balance, and enriched metabolic pathways, indicating HB1628’s potential in mitigating colitis and modulating gut health.	(258)

### 5.5 Kelch-like ECH-associated protein 1 (KEAP1) inhibitors used in IBD treatment

Therapeutic approaches targeting the KEAP1-NRF2 pathway primarily utilize KEAP1 inhibitors (259). These inhibitors block KEAP1 from binding to NRF2, resulting in the stabilization and activation of NRF2. Consequently, NRF2 activity increases, leading to the elevated expression of antioxidant enzymes such as glutathione S-transferase, NAD(P)H quinone oxidoreductase 1, and heme oxygenase-1 (260). These enzymes play a crucial role in reducing oxidative stress and inflammation in IBD. Currently, extensive studies are being carried out to explore the potential of KEAP1 inhibitors as treatments for IBD. For example, the intervention of KEAP1 inhibitors, such as natural coumarins, promotes Nrf2 activation, which reduces oxidative stress and inflammation in IBD by inhibiting NF-κB and enhancing antioxidant responses, as documented (261). Further studies on coumarin derivatives are essential for developing Nrf2 activators with intestinal anti-inflammatory activity. Similarly, CPUY192018, a potent small-molecule inhibitor of the Keap1-Nrf2 PPI, demonstrated cytoprotective effects in NCM460 colonic cells and a DSS-induced UC model by activating Nrf2 signaling (262). This suggests that direct inhibition of Keap1-Nrf2 PPI might be beneficial for UC treatment. By combining Keap1 inhibitors with H2S-donor moieties via molecular hybridization, DDO-1901 showed enhanced efficacy in alleviating colitis by mitigating oxidative stress and inflammation, outperforming parent drugs alone (263). Additionally, a recent research study investigates how 4-Octyl itaconate (OI), a form of itaconate functioning as a KEAP1 inhibitor, affects DSS-induced UC in mice (264). OI diminishes oxidative stress and cell death, boosts the gut barrier’s function, and lessens inflammation. It lowers the activity ofKEAP1, increases NRF2, and promotes the production of protective enzymes. This study highlights OI’s promising role in treating IBD. Hence, using KEAP1 inhibitors is crucial for treating IBD, where ROS plays a significant role.

## 6 Conclusion and future perspective

IBD represents a significant health challenge characterized by chronic inflammation of the digestive tract. The role of oxidative stress in the pathogenesis of IBD is well-documented, with high levels of reactive ROS and RNS contributing to gut mucosal damage and the activation of inflammatory pathways. Effective management of IBD may involve the use of antioxidants to mitigate oxidative stress, as evidenced by elevated oxidative stress markers such as malondialdehyde (MDA), 8-hydroxy-2’-deoxyguanosine (8-OHdG), and serum-free thiols (R-SH). Antioxidant therapies, including vitamin C, E, glutathione, and N-acetylcysteine, have shown the potential to alleviate IBD symptoms. Future research should focus on elucidating the detailed mechanisms by which oxidative stress contributes to IBD and exploring novel therapeutic strategies targeting this pathway. Specifically, targeting oxidative stress through molecular pathways such as MAPK, TLR4/NF-κB, Nrf2, and PI3K/Akt could offer new therapeutic avenues for IBD management. These pathways play critical roles in modulating inflammation and cellular responses to oxidative stress, providing promising targets for intervention. Polyphenol phytochemicals, such as curcumin, resveratrol, and others, have shown potential in modulating the molecular pathways, thereby reducing inflammation. These compounds exhibit antioxidant properties, neutralizing ROS and reducing oxidative stress, which is critical in the pathology of IBD. Further clinical trials are needed to validate these strategies’ effectiveness and establish standardized protocols for incorporating antioxidants into IBD treatment regimens.

## Author contributions

PM: Writing – original draft. LZ: Funding acquisition, Writing – review & editing. SL: Software, Writing – review & editing. ZZ: Software, Writing – review & editing. TJ: Writing – review & editing. FM: Conceptualization, Writing – review & editing. ZM: Writing – review & editing.
